# Genome sequence data of the contemporary fresh-market tomatoes

**DOI:** 10.1186/s12863-024-01249-3

**Published:** 2024-07-02

**Authors:** Juhee Kim, Tong Geon Lee

**Affiliations:** 1 Agriculture and Life Sciences Research Institute, Kangwon National University, Chuncheon, Republic of Korea; 2 Gulf Coast Research and Education Center, University of Florida, Wimauma, FL USA; 3 Horticultural Sciences Department, University of Florida, Gainesville, FL USA

**Keywords:** *Solanum lycopersicum*, Genetic variants, Nucleotide variability, Whole genome sequencing

## Abstract

**Objective:**

The fresh-market tomato (*Solanum lycopersicum*) is bred for direct human consumption. It is selected for specific traits to meet market demands and production systems, and unique genetic variations underlying fresh-market tomato yields have been recently identified. However, DNA sequence variant-trait associations are not yet fully examined even for major traits. To provide a rich genome sequence resource for various genetics and breeding goals for fresh-market tomato traits, we report whole genome sequence data of a pool of contemporary U.S. fresh-market tomatoes.

**Data description:**

Eighty-one tomatoes were nominated by academic tomato breeding programs in the U.S. Of the 81 tomatoes, 68 were contemporary fresh-market tomatoes, whereas the remaining 13 were relevant fresh-market tomato breeding and germplasm accessions. Whole genome sequencing (WGS) of the 81 tomatoes was conducted using the Illumina next-generation sequencing technology. The polymerase chain reaction (PCR)-free, paired-end sequencing libraries were sequenced on an average depth per sequenced base of 24 × for each tomato. This data note enhances visibility and potential for use of the more diverse, freely accessible whole genome sequence data of contemporary fresh-market tomatoes.

## Objective

Tomato (*Solanum lycopersicum*) is widely consumed worldwide [1], providing micronutrients in the human diet. The fresh-market tomato is one of the most consumed types of contemporary tomatoes [2], destined for fresh food ingredients such as tomato slice. There has been a significant improvement in the traits of the cultivated tomato, notably disease resistance and fruit quality [3]. Importantly, the fresh-market tomato is selected for distinguishable fruit traits (such as fruit size/shape/firmness), which are desirable in the industry (consumer market and production systems) [3]. However, the genetic architecture (characteristics of DNA sequence variations responsible for traits) in the contemporary fresh-market tomato needs to be further explored [4]. This is evidenced by the recent findings, 1) the positive effects from both previously known loci, most likely associated with tomato domestication and/or historical improvement, and new associations for fresh-market tomato yield and 2) the phenotypic variations for important, but not yet fully investigated, traits such as flavor in the contemporary fresh-market tomato germplasm [4]. Given this, exploiting DNA sequence variations responsible for traits has been of interest in the (applied) tomato research community. Furthermore, contemporary germplasm accessions are in high demand as such resources can be beneficial for rapidly incorporating favorable genotype combinations to achieve industry-driven traits (discussion in Bhandari et al. [4]). Whole genome sequence data had previously been used to identify the genetic recombination and DNA sequence variant-trait association in the contemporary fresh-market tomato [4–6], but sequence datasets (i.e., FASTQ files created using the Illumina platform) of previous studies were not published. To provide a rich genome sequence resource for future tomato research, we report the sequence datasets for 64 previously examined U.S. contemporary fresh-market tomatoes and 17 newly sequenced tomatoes.

## Data description

We selected 81 tomatoes that were nominated by academic tomato breeding programs located in Florida and North Carolina, major fresh-market tomato-producing areas of the U.S. (Table 1, Data file 1) [7]. Of the 81 tomatoes, 68 were contemporary fresh-market tomatoes, whereas the remaining 13 were relevant fresh-market tomato breeding and germplasm accessions. Of the 13 accessions, 12 accessions showed various plant architectures, such as a *brachytic* plant-like short architecture, and one accession (PI 128654) was known to carry an original Tomato spotted wilt virus resistance gene. 64 tomato sequence datasets (FASTQ files) were previously generated [4–6]. To further diversify the genetic resources of this tomato class, we sequenced the genomes of additional 17 tomatoes (14 contemporary and three relevant fresh-market tomato breeding and germplasm accessions; [8, 9]) using the same technical conditions and quality control as described in our previous study [4]. Plants were grown in the greenhouse as previously described [10], and the leaf tissue was collected approximately six weeks after sowing. Total genomic DNA was extracted from a single plant per each tomato using a DNeasy Plant Mini Kit (Qiagen). The PCR-free, paired-end libraries (DNA fragment length was 350 bp) were prepared from the extracted DNA, and sequenced using the Illumina next-generation sequencing technology. Illumina raw reads with adapter contamination and/or uncertain nucleotides constitute (Ns; > 10% of either read) were removed. Using the quality-controlled reads, we estimated genome coverage to be an average depth per sequenced base of 24 × [with Fla. 7060 and Micro-Tom showing the highest (31 ×) and lowest (5 ×) depths, respectively] (Table 1, Data file 1, Data set 1) [7, 11]. All sequenced tomatoes passed FastQC’s quality control (www.bioinformatics.babraham.ac.uk/projects/fastqc), both the mean quality score and per sequence quality score (Table 1, Data file 2) [12]. In addition, BWA (version 0.7.17; [13]) was used to map reads to the tomato reference genome sequence SL4.0 [14] to assess the mapping quality. The high mapping rate (> 98%) and mapping quality score (> 35) were calculated (Table 1, Data file 1) [7].

**Table 1:** Overview of data files/data sets

Label	Name of data file/data set	File types (file extension)	Data repository and identifier (DOI or accession number)
Data file 1	Tomato sequence note	Excel file (.xlsx)	figshare (https://doi.org/10.6084/m9.figshare.21799967) [7]
Data file 2	Tomato sequence FastQC	HTML file (.html)	figshare (https://doi.org/10.6084/m9.figshare.25499491) [12]
Data set 1	Genome sequence data of tomato	FASTQ file (.fastq)	NCBI Sequence Read Archive (https://identifiers.org/ncbi/insdc.sra:SRP484668) [11]

## Limitations

The current genome sequence data was generated using the Illumina next-generation sequencing technology. Some sequence data might have substantial gaps in coverage likely across complex regions of genetic variation if aligning reads to a fully sequenced reference genome is applied. (Phased) long-read sequence data coupled with fully sequenced DNA molecules such as bacterial artificial chromosome and Fosmid can be required in order to discover sequence variants in such gaps (for example, Oxford Nanopore and Illumina NovaSeq technologies used to sequence-resolve the Fusarium wilt resistance gene introgression in this fresh-market tomato class [15]).

## Data Availability

The data described in this Data note can be freely and openly accessed on NCBI Sequence Read Archive SRP484668 (Data set 1) and figshare Datasets 10.6084/m9.figshare.21799967 (Data file 1) and 10.6084/m9.figshare.25499491 (Data file 2). Please see Table 1 and references [7, 11, 12] for details and links to the data.

## Abbreviations

BWA: Burrows-Wheeler Aligner
PCR: Polymerase Chain Reaction
WGS: Whole Genome Sequencing
